# Enteroaggregative *Escherichia coli* in Daycare—A 1-Year Dynamic Cohort Study

**DOI:** 10.3389/fcimb.2016.00075

**Published:** 2016-07-13

**Authors:** Betina Hebbelstrup Jensen, Christen R. Stensvold, Carsten Struve, Katharina E. P. Olsen, Flemming Scheutz, Nadia Boisen, Dennis Röser, Bente U. Andreassen, Henrik V. Nielsen, Kristian Schønning, Andreas M. Petersen, Karen A. Krogfelt

**Affiliations:** ^1^Department of Microbiology and Infection Control, Statens Serum InstituteCopenhagen, Denmark; ^2^Department of Pediatrics, Copenhagen University Hospital HvidovreCopenhagen, Denmark; ^3^Department of Pediatrics, H.C. Andersen's Hospital, University of OdenseOdense, Denmark; ^4^Department of Clinical Microbiology, Copenhagen University Hospital HvidovreCopenhagen, Denmark; ^5^Department of Gastroenterology, Copenhagen University Hospital HvidovreCopenhagen, Denmark

**Keywords:** enteroaggregative *Escherichia coli*, EAEC, cohort study, carrier-state, daycare

## Abstract

Enteroaggregative *Escherichia coli* (EAEC) has been associated with persistent diarrhea, reduced growth acceleration, and failure to thrive in children living in developing countries and with childhood diarrhea in general in industrialized countries. The clinical implications of an EAEC carrier-status in children in industrialized countries warrants clarification. To investigate the pathological significance of an EAEC carrier-state in the industrialized countries, we designed a 1-year dynamic cohort study and performed follow-up every second month, where the study participants submitted a stool sample and answered a questionnaire regarding gastrointestinal symptoms and exposures. Exposures included foreign travel, consumption of antibiotics, and contact with a diseased animal. In the capital area of Denmark, a total of 179 children aged 0–6 years were followed in a cohort study, in the period between 2009 and 2013. This is the first investigation of the incidence and pathological significance of EAEC in Danish children attending daycare facilities. Conventional microbiological detection of enteric pathogens was performed at Statens Serum Institute, Copenhagen, Denmark, and at Hvidovre Hospital, Copenhagen, Denmark. Parents completed questionnaires regarding gastrointestinal symptoms. The EAEC strains were further characterized by serotyping, phylogenetic analysis, and susceptibility testing. EAEC was detected in 25 (14%) of the children during the observational period of 1 year. One or more gastrointestinal symptoms were reported from 56% of the EAEC-positive children. Diarrhea was reported in six (24%) of the EAEC positive children, but no cases of weight loss, and general failure to thrive were observed. The EAEC strains detected comprised a large number of different serotypes, confirming the genetic heterogeneity of this pathotype. EAEC was highly prevalent (*n* = 25, 14%) in Danish children in daycare centers and was accompanied by gastrointestinal symptoms in 56% of the infected children. No serotype or phylogenetic group was specifically linked to children with disease.

## Introduction

Enteroaggregative *Escherichia coli* (EAEC) is an established pathotype within the group of diarrheagenic *E. coli* (DEC), which also include enteropathogenic *E. coli* (EPEC), enterotoxigenic *E. coli* (ETEC), enteroinvasive *E. coli* (EIEC), and Shiga toxin-producing *E. coli* (STEC; Croxen et al., 2013). EAEC has been associated with cases of persistent diarrhea in children living in developing countries (Steiner et al., 1998), traveler's diarrhea (Adachi et al., 2001), and, most recently, cases of domestically acquired diarrhea in industrialized countries (Jenkins et al., 2006). Large outbreaks of EAEC-associated diarrhea have been reported in England (Dallman et al., 2014), Germany (Bielaszewska et al., 2011), Japan (Itoh et al., 1997), and South Korea (Shin et al., 2015). In the 2011 German outbreak, which affected thousands of Europeans, the EAEC strain had attained a prophage encoding a potent Shiga toxin, enabling the strain to cause hemolytic uremic syndrome, and bloody diarrhea, proving fatal in 54 cases (Bielaszewska et al., 2011).

The pathogenic properties of EAEC are under scrutiny, since EAEC can be isolated from asymptomatic carriers as well as from severe cases of diarrhea (Nüesch-Inderbinen et al., 2013; Tobias et al., 2015). EAEC exhibits substantial genetic diversity, and a wide range of virulence factors have been observed (Hebbelstrup Jensen et al., 2014). In addition, manifestations of disease due to EAEC are believed to depend on host factors (Jiang et al., 2003). Few studies have investigated the pathogenic potential of EAEC in the community in industrialized countries. In previous studies carried out in these settings, EAEC has mostly been associated with cases of travelers' diarrhea (Adachi et al., 2001). A high number of asymptomatic carriers of EAEC has been reported (Nüesch-Inderbinen et al., 2013), but to our knowledge, no study has investigated the clinical significance of long-term EAEC carriage in children in industrialized countries.

The aim of this study was to investigate the role of EAEC in childhood diarrhea in Danish daycare centers. To address this, we designed a cohort study of Danish children with a 1-year follow-up period per child. Exposure, transmission, and environmental factors were investigated in relation to EAEC carriage. Distinction between colonization and infection with EAEC relied on information provided by the children's parents in completed questionnaires. Further characterization of the EAEC strains was performed by serotyping and phylogenetic analysis with a view to identify strains potentially associated with gastrointestinal disease and clustering of EAEC strains specific to children in daycare.

## Materials and methods

### A cohort of healthy children attending daycare

Children aged 0–6 years attending municipal daycare centers in the Copenhagen metropolitan area were enrolled in a dynamic 1-year cohort study, as previously described (Hebbelstrup Jensen et al., 2016). Briefly, 179 children with a median age of 2 years participated in the study and were consecutively included in the period of 2009–2013. The parents of participation children provided written informed consent prior to enrollment into the study. The study was performed in accordance with the Declaration of Helsinki and was approved by The National Committee on Health Research Ethics in Denmark. Each child was observed for a 1-year period, and the parents were instructed to submit a stool sample from the child and answer a questionnaire every second month. The questionnaires included questions on symptoms including diarrhea (defined as three of more loose stools per day), exposures (e.g., intake of contaminated food or water), contact with another individual suffering from diarrhea, contact to diseased animals, and recent travel, which was defined as any trip outside of Denmark in a period of 2 months prior to sampling. Long-term carriage of EAEC was defined as testing positive for EAEC in stool samples for 6 months or longer, which has been defined similarly in other studies (Richardson et al., 1981; Lübbert et al., 2014; Ismail et al., 2016). Gastrointestinal symptoms reported included diarrhea, abdominal pain, vomiting, nausea, weight loss, and reduced appetite. An episode with the given symptom was registered, when reported by the parents.

### Microbiological analyses

Conventional microbiological tests for enteric viruses, bacteria, and parasites of potential clinical relevance were performed, including rotavirus, adenovirus, norovirus, sapovirus, *E. coli* spp., *Salmonella* spp., *Clostridium difficile, Campylobacter* spp., *Yersinia* spp., *Shigella* spp., *Vibrio* spp., *Aeromonas* spp., *Giardia intestinalis, Cryptosporidium* spp., *Entamoeba histolytica, Entamoeba dispar, Dientamoeba fragilis, Blastocystis*, and *Ascaris* spp. (Blom et al., 1999; Verweij et al., 2004, 2007; Logan et al., 2006; Oka et al., 2006; Persson et al., 2008, 2015; van Maarseveen et al., 2010; Stensvold and Nielsen, 2012). Of the 719 stool samples, 688 were available for DNA extraction (Mirsepasi et al., 2014) and quantitative PCR analysis for enteric parasites (Verweij et al., 2004, 2007; Stensvold and Nielsen, 2012) and viruses (Logan et al., 2006; Oka et al., 2006; van Maarseveen et al., 2010), in the remaining 31 cases the submitted sample material was insufficient for analysis. From bacterial cultures, at least five *E. coli* colonies of different morphology were handpicked, and identification of diarrheagenic *E. coli* isolates was performed by PCR targeting the ETEC, STEC, EIEC, and EPEC genes (Persson et al., 2007). The EPEC pathotype includes attaching and effacing *E. coli* (AEEC), which is defined by the presence of the *eae* (intimin) gene, but which lacks the conventional serotypes belonging to the EPEC pathotype (Persson et al., 2007).

To diagnose EAEC, the genes *aatA, aggR*, and *aaiC* were targeted as previously described (Boisen et al., 2008); detection of at least one of these genes was considered diagnostic of EAEC. EAEC strains were further characterized by DNA probe hybridization targeting the genes *aatA, aggR, aaiC*, and *astA* (Baudry et al., 1990) and by O:H serotyping, using the method developed by Orskov and Orskov (1992). Antimicrobial susceptibility testing was performed using the disk diffusion method according to the guidelines of the Clinical Laboratory Standards Institute CLSI (2011), where the 0.5 McFarland standard on Müeller–Hinton II agar plates (BBLTM, US) was used. The antibiotics tested for included mecillinam, piperacillin-tazobactam, cefoxitin, cefotaxime, ampicillin, ciprofloxacin, nalidixic acid, trimethoprim, sulfamethoxazole, gentamicin, tetracycline, and nitrofurantoin. The results of the susceptibility tests were assessed with reference to the EUCAST breakpoints as stipulated by the CLSI guidelines (Clinical Laboratory Standards Institute). Multi-drug resistance was defined as acquired resistance to ≥3 antibiotics from different groups (Magiorakos et al., 2012). Classification of EAEC strains was based on the genes *TspE4.C2, chuA*, and *yjaA* by PCR, which enables division of the EAEC strains into the phylogenetic groups A, B1, B2, and D as previously described (Clermont et al., 2000).

### Statistics

To assess if multi-drug resistant EAEC strains were isolated more frequently in children with reports of traveling we compared the proportions of multi-drug resistant strains collected from children with and without reports of traveling. The Fishers exact test was used to estimate a possible statistical significant difference for detection of multi-drug resistance in EAEC strains collected from children with reports of recent travel.

To estimate the relative risk of testing positive for EAEC for children, who had traveled outside of Denmark, we calculated the risk of testing positive for EAEC for children who had traveled (in the nominator) divided by the risk of testing positive for EAEC in children, who did not have reports of traveling (in the denominator).

The Statistical software for windows SAS® version 9.4 (SAS institute Inc., Cary, NC, USA) was used for data analysis. The study was approved by the Danish Data Protection Agency [protocol number 2013-41-2338].

### Ethics

The study was performed in accordance with the Declaration of Helsinki and was approved by The National Committee on Health Research Ethics, [protocol number H-A-2008-111].

## Results

During the cohort study, 719 stool samples were collected from 179 children healthy enough to attend daycare; on average four stool samples were submitted from each child. In total, 143 cases of diarrhea were reported by parent-based assessment. The 143 diarrheal episodes were distributed among 90 children. Other gastrointestinal symptoms included vomiting (143 episodes), abdominal pain (144 episodes), loss of appetite (138 episodes), nausea (67 episodes), and/or weight loss (27 episodes). In total, other gastrointestinal symptoms were reported 519 times, affecting 146 children (82%).

EAEC was detected in 32 stool samples (4%). In total, 37 EAEC strains were isolated from 25 (14%) children in the cohort (Table 1), where five samples were positive for two different EAEC strains. The genetic profiles of EAEC strains collected were confirmed by DNA hybridization with a 100% match. EAEC was frequently detected, second in incidence only to AEEC. One or more gastrointestinal symptoms were reported within a period of 2 months prior to sampling by 14 (56%) of the 25 children positive for EAEC. The most frequently reported EAEC-associated symptoms included reduced appetite and vomiting (32%), followed by diarrhea (24%), and abdominal pain (16%). The median duration of diarrhea in EAEC cases (*n* = 6) was 2 days. Recent travel was reported by 36% of the EAEC-positive children. Only one EAEC-positive child had been treated with antibiotics prior to sampling. The distribution of additional enteric pathogens detected among EAEC-positive children is presented in Table 2.

**Table 1:** **Characterization of EAEC strains solated in the study**.

**Daycare no**.	**Child**	**Age in years**	**Sample no.(1-6)**	**Sample ID**	**Serotype**	**EAEC genes^a^**	**Phylogenetic group**	**Symptoms reported**	**Foreign travel^b^/destination**
1	A	3	3	C 506-10-1115^c^	O111:H21	*aatA, aggR, aaiC*	B1	Diarrhea Abdominal pain	
2	B	1	1	C 135-10-1029^d^	O86:H30	*aatA, aggR*	D	Vomiting Reduced appetite	Norway
3	C^e^^f^	3	1	C 198-10-1076	O3:H2	*aatA, aggR, aaiC*	A	Reduced appetite	
			3	C511-10-1175	O3:H2	*aatA, aggR, aaiC*	A	None	
			5	C 72-11-1284	O3:H2	*aatA, aggR, aaiC*	A	Vomiting Reduced appetite	
4	D	4	4	C 876-10-1231^d^	O111:H21	*aatA, aggR, aaiC*	B1	Diarrhea Vomiting Reduced appetite	
5	E^g^	4	3	C 896-10-1202	O99:H4	*aatA, aggR, aaiC*	A	None	
5	F^g^	2	3	C 380-14-1203	O99:H4	*aatA, aggR, aaiC*	A	None	
5	G	7	5	C 74-11-1288-A^i^	O92:H33	*aatA, aggR, aaiC*	A	Abdominal pain reduced appetite	
				C 75-11-1288-B	O92:H33	*aatA, aggR, aaiC*	B1		
5	H	6	5	C 77-11-1289-A^i^	O?:H-	*aatA*	A	Diarrhea Abdominal pain Reduced appetite	
				C 78-11-1289-B	Orough:H-	*aatA*	B1		
6	I	1	5	C 25-11-1270	O127:H21	*aatA, aggR, aaiC*	A	N/A	
7	J^h^	2	2	C 880-10-1242^d^	O78:H2	*aatA, aggR, aaiC*	A	Vomiting	Sweden
7	K^h^	5	2	C 884-10-1241^d^	O78:H2	*aatA, aggR, aaiC*	A	None	Sweden
8	L	1	6	C 831-11-1405	O92:H33	*aatA, aggR, aaiC*	A	Diarrhea Reduced appetite	Macedonia
9	M	3	6	C 707-13-1630-A^i^	O14:H-	*aatA, aggR, aaiC*	A	N/A	
				C 27-13-1630-B	(O48), O62:H-	*aatA, aggR, aaiC*	B1		
10	N	6	1	C 409-12-1496	O127:H21	*aatA, aggR, aaiC*	B1	None	
11	O	5	1	C 404-12B-1446	O15:H-	*aatA, aggR, aaiC*	A	N/A	
			6	C 725-13-1750^c^	O14:H-	*aatA*	A	Diarrhea Nausea Abdomin al pain	
12	P	2	1	C 249-13-1451	O86:H2	*aatA, aggR, aaiC*	A	None	N/A
			4	C 31-13-1641	O17:H18	*aaiC*	D	None	Turkey
13	Q	3	1	C 407-12-1453^c^	O44:H18	*aatA, aggR, aaiC*	D	None	
14	R	1	1	C 250-13-1460^d^	O9:H21	*aatA, aggR, aaiC*	B1	Diarrhea Vomiting	Tanzania
15	S	4	2	C 383-14-1508^d^	O114:H21	*aaiC*	A	None	
			3	C 1028-12-1564^d^	O114:H32	*aaiC*	A	Vomiting	
15	T	2	2	C 1030-12-1570-A^c^^d^^i^	O23:H-	*aatA, aggR, aaiC*	B1	None	
				C 1031-12-1570-B^c^	O55:H10	*aatA, aggR, aaiC*	B1		
15	U	2	5	C 35-13-1659	O103:H43	*aatA*	A	None	Turkey
16	V	1	2	C 413-12-1554^d^	O153:H30	*aatA, aggR*	D	Vomiting	
17	W^j^	2	3	C 374-14-1618^d^	O?:H34	*aatA, aggR, aaiC*	D	None	Seychelles
			4	C 34-13-1657&^d^	O?:H34	*aatA, aggR, aaiC*	D	Nausea Vomiting	
			6	C 724-13-1748^d^	O?:H34	*aatA, aggR, aaiC*	D	None	
19	X	2	5	C 73-13-1690-A^d^^i^	O?:H30	*aatA, aggR*	D	Reduced appetite	Egypt
				C 719-13-1690-Bd	O153:H30	*aatA, aggR*	D		
20	Y	N/A	N/A	C 382-14-1422	O125:H21	*aatA, aggR, aaiC*	B1	N/A	N/A

*Abbreviations: EAEC, enteroaggregative Escherichia coli, aatA, gene encoding the dispersin transporter protein, aggR, gene encoding a transcriptional activator, aaiC, gene encoding a secreted protein*.

a *Detected by PCR and confirmed by DNA hybridization*.

b *Foreign travel reported within the last 2 months prior to stool sampling*.

c *Co-infection with AEEC*.

d *Multi-drug-resistant EAEC strain*.

e *Carrier of the same EAEC strain for a year*.

f *Use of antibiotics*.

g *Siblings*.

h *Sibling*.

i *Infected with two different EAEC strains at the same point of observation*.

j *Carrier of the same EAEC strain for 10 months*.

*N/A not available*.

**Table 2:** **Co-infections among the 35 EAEC-positive cases**.

**Co-infection**	**EAEC-positive cases**	**Diarrheal cases**	**Cases with vomiting**	**Undefined**
Blastocystis	1	0	0	0
*Dientamoeba fragilis*	26	4	6	4
Norovirus genotype I	2	1	1	0
Norovirus genotype II	5	0	1	1
Sapovirus	3	0	0	0
Adenovirus	1	0	0	1
AEEC	4	2	1	0
Aeromonas spp.	1	0	0	0
None	5	3	1	0

*EAEC, enteroaggregative Escherichia coli; AEEC, attaching, and effacing Escherichia coli, D. fragilis, Dientamoeba fragilis*.

*Co-infections in EAEC-positive samples were distributed as follows: D. fragilis (14 cases), D. fragilis, and AEEC (4 cases); Norovirus genotype II (1 case), D. fragilis and norovirus genotype I (1 case); D. fragilis and norovirus genotype II (1 case), D. fragilis and sapovirus (2 cases); D. fragilis and adenovirus (1 case); D. fragilis, Blastocystis and Norovirus genotype II (1 case), D. fragilis, Aeromonas spp, and norovirus genotype II (1 case); D. fragilis, sapovirus, norovirus genotype I and norovirus genotype II (1 case)*.

### EAEC serotyping

**Figure 1:**
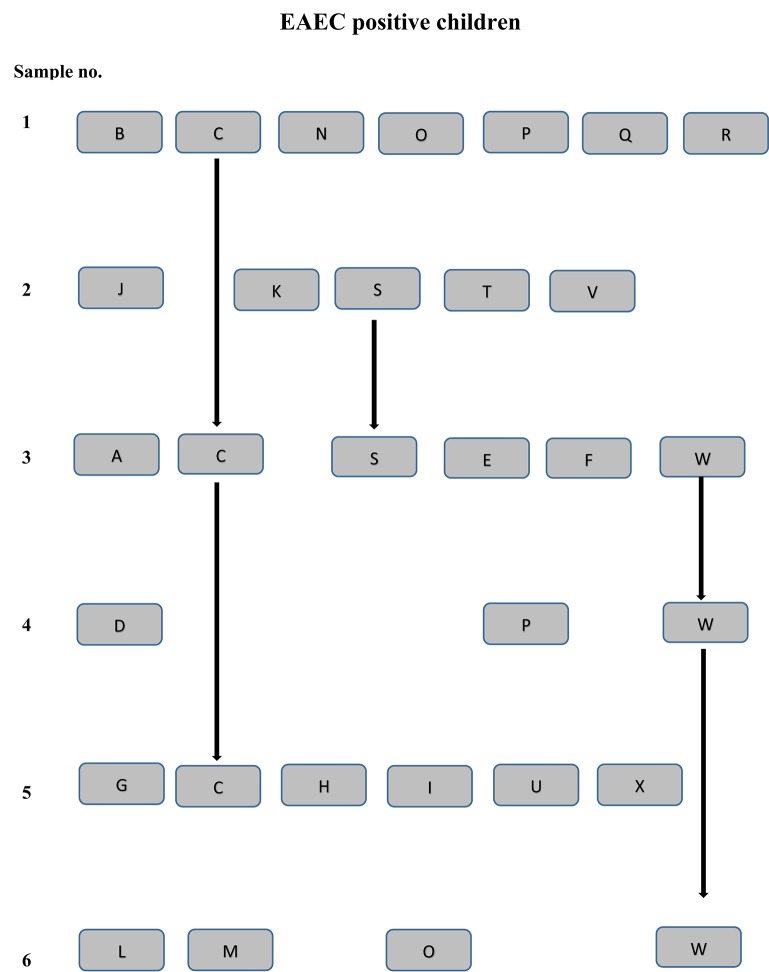
**EAEC positive children are shown for each sampling point (1–6) by a unique letter (A–X)**. The lines connecting the letters indicate EAEC strains of the same serotype collected at several sampling points. Children by the letters P and O tested positive for EAEC at several sampling point, but with EAEC strains of different serotypes, genetic profile, and resistance pattern. Child Y tested positive for EAEC, but is not included in the figure since the sampling point was unknown for this child.

The EAEC strains were seen to comprise a wide range of serotypes. Two pairs of siblings were each colonized with EAEC strains of the serotypes O99:H4 and O78:H2, respectively. Both pairs of the EAEC strains belonged to the phylogenetic group A, and had the EAEC virulence gene profile, *aggR, aatA*, and *aaiC* (Table 1). Likewise, five pairs of children cared for in 10 different institutions were colonized with EAEC strains of identical serotypes, O111:H21, O14:H-, O92:H33, O127:H21, and O153:H30, respectively. For each set of strains with matching serotypes, the samples were submitted within the same year. However, the two EAEC strains of serotype O127:H21 belonged to different phylogenetic groups, B1 and A, respectively. The two EAEC strains of serotype O14:H- possessed different EAEC genes; one strain exhibited the *aatA* gene, while the other strain tested positive for the *aggR, aaiC*, and *aatA* genes. No common source of exposure could be identified for the three pairs of children colonized with EAEC strains with identical serotype, phylogenetic group, and EAEC genetic profile (Figure 1).

### Multi-drug resistant EAEC strains

**Figure 2:**
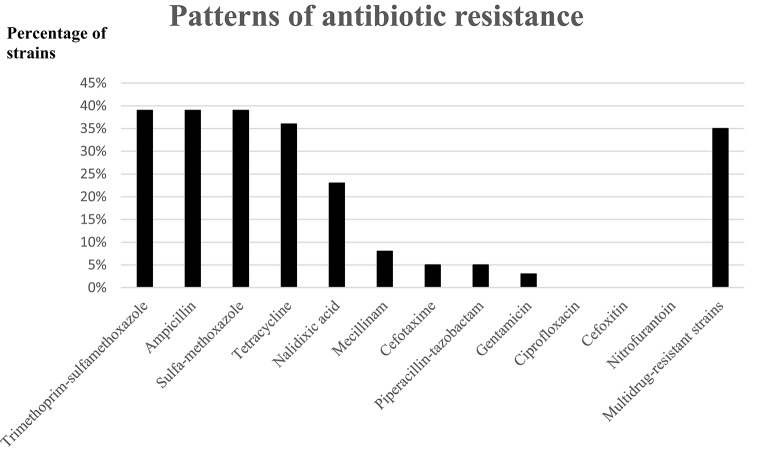
**Patterns of antibiotic drug resistance in the EAEC strains**.

Multi-drug resistance was observed in 13 (35%) of the EAEC strains collected (Figure 2). Multi-drug resistant EAEC strains were not overrepresented in children with reports of recent travel, when compared with children without reports of recent travel, (*n* = 5, 38 vs. *n* = 8, 62%) *p* = 0.69 (Figure 2). Resistance toward ciprofloxacin, cefoxitin, or nitrofurantoin was not detected in any of the strains.

### Co-infections among EAEC-positive children

Co-infection with one or more microorganisms was frequently observed for the EAEC-positive children. Twenty-one EAEC-positive children had co-infections at 27 observational points (Table 2). The protozoan *D. fragilis* was the most commonly co-infecting organism among the EAEC cases and was observed in 21 EAEC-positive children at 26 observational points. Norovirus genotype II was detected in five EAEC-positive children and AEEC in four children. Other co-infections included sapovirus (three children) and norovirus genotype I (two children). Adenovirus, *Aeromonas* spp., and *Blastocystis* were detected in one EAEC-positive child each.

### Risk factors and exposures among EAEC cases

In total, foreign recent travel was reported for 77 children in the cohort, of whom nine (12%) tested positive for EAEC. However, testing positive for EAEC was not associated with any history of recent travel within the preceding 2 months (RR, 0.87; 95% confidence intervals, 0.63–1.20). Contact with a diseased animal was reported 25 times in the study, but only by two (8%) children tested positive for EAEC. Contact with another individual suffering from diarrhea was reported in 42 cases of diarrhea, with only two (5%) of them being EAEC-positive cases. No additional testing for EAEC was performed on these contacts. Intake of suspected contaminated food or water was not reported by any of the EAEC-positive cases.

### EAEC long-term carriage

Long-term carriage of EAEC was observed for two children. Persistent diarrhea, weight loss, or other signs of failure to thrive were not reported with long-term carriage. However, reduced appetite and nausea were reported within EAEC-positive episodes. One child with long-term EAEC carriage was positive for EAEC upon inclusion in the study, and the primary infection with EAEC may have occurred prior to the study period. The other child tested positive in samples nos. 3 through 6, and EAEC colonization may have persisted beyond the study period. Only four children did not submit a consecutive sample after testing positive for EAEC. Two of the children dropped out of the study and the remaining two children had submitted their final sample. Further, evaluation of the EAEC carrier statuses for these children was not performed.

## Discussion

Previously, EAEC has been linked to conditions such as failure to thrive and reduced growth acceleration in children living in developing countries (Steiner et al., 1998). In this longitudinal study of Danish children in daycare centers, EAEC was only linked to diarrhea in 6/143 (4.2%) diarrheal cases (Table 1). Diarrhea, failure to thrive, or weight loss was not associated with EAEC carriage. These findings indicate that the nutritional status might play an important role in the clinical manifestation of EAEC infections, which has been demonstrated in a mouse model of EAEC pathogenicity (Roche et al., 2010). Another Danish case-control study of domestically acquired childhood diarrhea failed to associate EAEC with diarrhea in children < 5 years (Olesen et al., 2005). In that study, EAEC was detected by the CVD432 probe, and 39.9% of the children with diarrhea were cared for in private daycare, (Olesen et al., 2005) where the children are less exposed to pathogens compared with the children included in our study (Zutavern et al., 2007). In our study, gastrointestinal symptoms were reported by 56% of the EAEC positive children (Table 1); however, a high level of co-infection was observed among the EAEC cases (Table 2).

Asymptomatic EAEC carriage has been reported in a large number of children living in developing countries (Boisen et al., 2012). This phenomenon has been proposed to be caused by acquired immunity due to frequent exposure to EAEC, as well as colonization rather than infection. Asymptomatic carriage of a conventional enteric pathogen is exemplified by *G. intestinalis* (Bartlett et al., 1985), which has been detected in 19% (*n* = 105) of asymptomatic children in daycare. Another example is *Campylobacter* spp. (Richardson et al., 1981), which was isolated from six healthy South African school children in a period of 9 months and for more than a year for three children.

Serotyping of EAEC strains is highly useful in outbreaks, due to the considerable genetic diversity observed among EAEC strains (Jenkins et al., 2006). Various serotypes of EAEC strains have previously been reported in sporadic cases of diarrhea unrelated to specific outbreaks (Boisen et al., 2012) and detection of similar EAEC serotypes within a group of individuals is usually interpreted as an exposure to a common source of infection. Two EAEC strains of the serotypes O99:H4 and O78:H2 were each detected in two pairs of siblings within the same sampling period, suggesting exposure to a common source of infection or that the transmission of EAEC may primarily occur among children in close proximity. Interestingly, three pairs EAEC strains of the same serotypes (O111:H21, O92:H33, and O153:H30) were detected in three pairs of children from six different daycare centers (Table 1). Any common source of infection with EAEC could not be determined for these children.

Several of the serotypes found in this study have previously been described in EAEC-positive children. One example is a study performed in Mali, where EAEC strains of serotype O14:H- were found in a child without diarrhea, serotype O15:H- was observed in a child with diarrhea, and EAEC strains of serotypes O86:H30 and O153:H30 were isolated from both cases and controls (Boisen et al., 2012). EAEC serotype O99:H4 has previously been detected in a 4-year-old with acute diarrhea with a recent history of traveling to India (Huppertz et al., 1997) and in infants with acute diarrhea in Brazil (Moreno et al., 2010). Serotype O127:H21 has been associated with children suffering from diarrhea in Egypt (Behiry et al., 2011), and in a Brazilian child with diarrhea (Moreno et al., 2010). Of particular interest is the isolation of three of the five serotypes defining the prototype strains for EAEC; 042 serotype O44:H18, 17-2 serotype O3:H2, and JM221 serotype O92:H33 (Mathewson et al., 1986; Nataro et al., 1987). These serotypes have been isolated from studies performed in Peru, Chile, and Mexico, respectively. From these serotypes, only serotype O92:H33 was collected from a child with diarrhea in our study. In 2006, a foodborne outbreak of gastroenteritis in Italy was associated with EAEC serotype O92:H33 (Scavia et al., 2008) and was detected in a child in the present study, who had traveled to Macedonia, which is geographically close to Italy. The EAEC strain of serotype O?:H- was recently associated with a Korean outbreak of foodborne gastroenteritis in children (Shin et al., 2015). Finally, serotype O111:H21 was recently isolated from a case of hemolytic uremic syndrome in Northern Ireland; however, in this case, the EAEC strain was infected by a bacteriophage encoding the Shiga toxin (Dallman et al., 2012), which indicates a pathogenic potential of this serotype beyond that of EAEC only. These findings highlight the usefulness of serotyping EAEC strains and should encourage further characterization of EAEC strains with a view to elucidating the relations and origins of EAEC.

Two children were carriers of EAEC strains of the same serotype (O3:H2 and O?:H34) for several months, highlighting the colonization potential of EAEC. One of the strains observed in long-term carriage in this study exhibited the same serotype as EAEC reference strain 17-2 (O3:H2; Nataro et al., 1995). Since this EAEC strain was identified in the first stool sample submitted from the child, it is unknown whether initial inoculation had caused diarrhea. In another child, serotype O?:H34 was detected in the third sample submitted, but no episode of diarrhea was reported at this point of observation.

Our study adds support to the considerable genetic diversity previously seen among EAEC strains isolated from children with and without gastrointestinal symptoms. No serotype or phylogenetic group was limited to children with or without gastrointestinal symptoms. Other studies have failed to associate a specific EAEC phylogenetic trait or virulence factor with disease, including a Nigerian case-control study (Okeke et al., 2010), where phylogenetic groups and MLST types were scattered among cases and control. A Brazilian case-control study, (Regua-Mangia et al., 2009), also failed to identify a statistically significant association between EAEC virulence traits, or EAEC serotypes and disease. This lack of association between virulence factors and disease has been demonstrated for other pathotypes, such as the STEC (Ferdous et al., 2016).

Co-infection with EAEC and enteric pathogens has been described frequently especially in cases of travelers' diarrhea and in childhood diarrhea (Cohen et al., 2005). A high number of co-infections has been proposed to reflect common exposure to enteropathogens as well as improved microbiological diagnostics (Zboromyrska et al., 2014). In this study, 20 of 25 EAEC-positive children were co-infected. Several of the co-infections in EAEC positive children with gastrointestinal symptoms were with AEEC and the pathogenicity of AEEC has not been well-established in Danish children; in a case-control study AEEC was detected more frequently in cases compared with controls (Olesen et al., 2005). Furthermore, the pathogenicity of *D. fragilis* in Danish children has recently been questioned in a randomized control trial (Röser et al., 2014). However, the level of co-infection among EAEC cases makes the interpretation of the gastrointestinal symptoms reported in our study difficult and is a limitation in our study. However, only three from six children with diarrhea and EAEC were co-infected.

A high level of multi-drug resistance was detected among the EAEC strains (35%), but all strains were susceptible to nitrofurantoin and ciprofloxacin. A high level of resistance toward ampicillin (39%) was observed (Figure 2). The observed level of multi-drug resistance among the EAEC strains collected in Danish children in daycare is an issue of concern. Investigation of the level of antibiotic resistance in daycare is of socio-economic interest since transmission of enteric pathogens from daycare into the community have been reported (Sullivan et al., 1984; Venczel et al., 2001; Sacri et al., 2014).

Previously, we have presented the same cohort of children, where only the epidemiological data were available; we found a trend toward an increased risk of developing diarrhea in children with a history of infant colic, low birth weight, and to a lesser extent, children who had consumed antibiotics (Hebbelstrup Jensen et al., 2016). Of the EAEC positive children, only one had reports of antibiotic use, and none had suffered from infant colic or was of low birth weight.

Measurement of antibodies toward different EAEC virulence genes has previously shown that the presence of such antibodies does not protect against re-colonization (Bellini et al., 2005). In addition, several host factors such as genetic polymorphisms in the promoter region of the IL-8 gene (Jiang et al., 2003) have been shown to predispose to EAEC-induced disease. These factors combined may play explain the different manifestations of disease observed in the EAEC-positive children in our cohort.

## Conclusion

EAEC is highly prevalent among Danish children in daycare and was detected in 4% of diarrheal cases in the cohort. Gastrointestinal symptoms were not associated with any EAEC serotype or phylogenetic group. Long-term carriage of EAEC was observed for two children without failure to thrive or weight loss. The high level of multi-drug resistance observed in EAEC strains in Danish children in daycare is a cause for concern.

## Author contributions

KK, AP, CS, and HN obtained the funding for this research and designed the study. DR and BA performed the inclusion and enrollment of study participants. NB, KS, KO were responsible for the microbiological analyses performed in the study. KS, BJ, CS, CRS, KK, and HN were responsible for the interpretation of the research data and of the microbiological analyses. All authors have contributed to the writing of the manuscript and have all approved the final draft of the manuscript. All authors take responsibility for the accuracy and integrity of the research performed.

## Funding

This work was supported by The Danish Council for Strategic Research, Innovation and Higher Education [grant number 2101-07-0023] granted to Professor Karen Angeliki Krogfelt.

### Conflict of interest statement

The authors declare that the research was conducted in the absence of any commercial or financial relationships that could be construed as a potential conflict of interest.
